# MnTBAP Therapy Attenuates Renal Fibrosis in Mice with 5/6 Nephrectomy

**DOI:** 10.1155/2016/7496930

**Published:** 2016-02-17

**Authors:** Jing Yu, Song Mao, Yue Zhang, Wei Gong, Zhanjun Jia, Songming Huang, Aihua Zhang

**Affiliations:** ^1^Department of Nephrology, Nanjing Children's Hospital, Nanjing Medical University, Nanjing 210008, China; ^2^Jiangsu Key Laboratory of Pediatrics, Nanjing Medical University, Nanjing 210029, China; ^3^Nanjing Key Laboratory of Pediatrics, Nanjing 210008, China

## Abstract

Renal fibrosis is a common pathological feature of all kinds of chronic kidney diseases (CKDs) with uncertain mechanisms. Accumulating evidence demonstrated an important role of oxidative stress in the pathogenesis of CKD. Here we hypothesized that MnTBAP (manganese (III) tetrakis (4-benzoic acid)porphyrin chloride), a cell-permeable mimic of superoxide dismutase (SOD), may protect against the fibrotic response in CKD by antagonizing oxidative stress. To verify this hypothesis, we performed experiments in tubular epithelial cells and mice with 5/6 nephrectomy (Nx). In mouse tubular epithelial cells, TGF-*β*1 induced a significant transition to fibrotic phenotype in line with a remarkable mitochondrial dysfunction, which was markedly improved by MnTBAP (1.14 *μ*M) pretreatment. In remnant kidneys of 5/6 Nx mice, tubulointerstitial fibrosis occurred in parallel with mitochondrial abnormality in renal tubular cells. Administration of MnTBAP significantly attenuated the deposition of extracellular matrix as evidenced by the blocked expressions of fibronectin, collagen I, and collagen III. Masson staining also displayed an ameliorated accumulation of collagenous matrix in MnTBAP-treated mice. Moreover, MnTBAP also significantly improved the severity of proteinuria without altering CKD-related hypertension. Collectively, MnTBAP therapy served as a promising strategy in preventing renal fibrosis in CKDs possibly via antagonizing mitochondrial-derived oxidative stress and subsequent protection of mitochondrial function.

## 1. Introduction

Chronic renal failure (CRF) is the common end stage of all kinds of CKDs. Currently, there are no effective treatments to prevent the progression of CKD to CRF in the clinic [1, 2]. During the progression of CKDs, renal fibrosis is a common feature and contributes to the local inflammation and permanent loss of renal function [3]. Thus exploration of pathogenic mechanisms of renal fibrosis and defining some novel antifibrotic therapies are becoming urgent and important.

Chronic pathological insults cause phenotypic alteration in renal tubular cells [4], resulting in the loss of epithelial cell-cell-basement membrane contacts and structural/functional polarity and the acquisition of a fibroblastic phenotype, thus contributing to the renal fibrosis [5, 6]. Roles of the TGF-*β* superfamily in renal fibrogenesis have been extensively studied. Among the members of TGF-*β* superfamily, TGF-*β*1 is the most potent profibrogenic cytokine. It also plays an important role in modulating inflammation and cell infiltration in diseased kidney, which subsequently promotes renal fibrosis [7, 8]. TGF-*β*1 inhibition has been considered as a possible antifibrotic therapy in CKDs. However, the multiple functions of TGF-*β*1 in regulating physiology and pathology beyond fibrosis greatly limited the application of the therapies targeting TGF-*β*1 signaling [9, 10].

Mitochondria are the important intracellular organelles responsible for vital cellular activities such as energy production, ROS generation, and regulation of cell death pathways. As an organ with huge energy demand, kidney has abundant mitochondria in all kinds of resident cells. Mitochondrial dysfunction would cause oxidative stress, inflammation, and subsequent cell damage and fibrosis [11, 12]. It has been reported that mitochondrial complex I inhibitor rotenone protected kidneys against obstructive injury possibly via inhibiting mitochondrial oxidative stress, inflammation, and fibrosis [13]. In fact, mitochondria themselves can be the first injury target of mitochondria-derived ROS, forming positive pathogenic cross talk between mitochondrial oxidative stress and mitochondrial impairment.

In the present study, using a pharmacological strategy, we examined the role of MnTBAP, a synthetic SOD mimic, in modulating fibrogenesis in a CKD model of 5/6 Nx being a most relevant animal model to mimic human CKDs. The findings from the current study not only provided more evidence showing the mitochondrial role in mediating renal fibrosis in CKDs but also offered potentials of MnTBAP targeting mitochondrial ROS in treating CKDs.

## 2. Materials and Methods

### 2.1. Reagents and Antibodies

Antibodies of anti-E-cadherin, anti-*α*-SMA, anti-vimentin, anti-fibronectin, anti-collagen I, and anti-collagen III were purchased from Abcam (Cambridge, MA). Antibodies of anti-GAPDH and the secondary antibody were bought from Bioworld (Nanjing, China). TGF-*β*1 and MnTBAP were from Sigma (USA).

### 2.2. Cell Culture

DMEM-F12 medium and newborn bovine serum were purchased from Wisent Corporation (Wisent, Canada). Immortalized mouse proximal tubular cells (mPTCs) from Sciencell Research Laboratory (Cat #: M4100) were maintained at 37°C with 5% CO_2_. The cells were subcultured using 0.25% trypsin-0.02% EDTA at 70–80% confluence (Invitrogen, USA). Cells were treated with TGF-*β*1 at a concentration of 10 ng/mL to induce phenotypic transition with or without a pretreatment of MnTBAP (1.14 *μ*M).

### 2.3. Animal Studies

C57BL/6J mice were purchased from Shanghai SLAC Laboratory Animals (Shanghai, China). All animals were housed under conventional conditions with controlled temperature, humidity, and light (12 h light-dark cycle) and were provided a standard commercial diet and water. 5/6 Nx was performed through two stages as described previously [14]. In detail, the left kidney was decapsulated via left flank incision to resect the upper and lower poles. One week later, the right kidney was removed via right flank incision. MnTBAP is dissolved in physiological saline solution with pH 7.0. Mice were treated with MnTBAP at a dose of 10 mg/kg by intraperitoneal injection (three times per week) after the second surgery. The control animals were injected with the same volume of physiological saline solution. Twelve weeks later, systolic blood pressure was measured by computerized tail-cuff system (BP-2000, Visitech Systems, Apex, NC, USA). The urine was collected and analyzed for the urine albumin and creatinine by using commercial EIA kits. Mice were anesthetized with an intraperitoneal injection of a ketamine/xylazine/atropine mixture, and the renal tissues were harvested and fixed for Masson trichrome staining or frozen immediately in liquid nitrogen and stored at −80°C for further analysis. All procedures were in accordance with the guidelines approved by the Institutional Animal Care and Use Committee at Nanjing Medical University (number 20090053).

### 2.4. Quantitative Real-Time-PCR (qRT-PCR)

Total RNA from cultured mPTCs and renal tissues were extracted by TRIzol reagent (Invitrogen, Carlsbad, CA) according to the manufacturer's instructions. Reverse transcription was performed using Transcriptor First Strand cDNA Synthesis Kit (Takara, Japan) according to the manufacturer's instruction. qRT-PCR was used to detect the target gene expression. The qRT-PCR was performed using the SYBR Green Master Mix on the ABI Prism 7500 Sequence Detection System (Foster City, USA). The relative gene expression level was calculated through delta-delta Ct method and GAPDH was used as the internal control. Experiments were repeated in triplicate.

### 2.5. Western Blotting

mPTCs and renal tissues were lysed in RIPA buffer (Biyuntian, Shanghai, China) containing the protease inhibitor PMSF. Lysates were separated by 10% SDS-PAGE. Immunoblotting was performed with primary antibodies anti-E-cadherin (1 : 1000), anti-*α*-SMA (1 : 1000), anti-vimentin (1 : 1000), anti-fibronectin (1 : 1000), anti-collagen I (1 : 1000), anti-collagen III (1 : 1000), and anti-GAPDH (1 : 1000). The blots were visualized with Amersham ECL Detection Systems (Amersham, Buckinghamshire, UK). Densitometric analysis was performed using Imagelab Software (Bio-Rad, USA).

### 2.6. Analysis of ROS Production, Mitochondrial Membrane Potential (MMP), and mtDNA Copy Number

2′,7′-Dichlorofluorescein diacetate (DCFDA) was used to measure intracellular ROS production in mPTCs. To examine ROS levels, DCF fluorescence was analyzed by flow cytometry at 485 nm excitation and 528 nm emission. The mitochondrial MMP in mPTCs was determined using the lipophilic cationic probe 5,5′,6,6′-tetrachloro-1,1′,3,3′-tetraethyl-benzimidazol-carbocyanine iodide (JC-1; Molecular Probes). JC-1 dye exhibits potential-dependent accumulation in mitochondria, indicated by a fluorescence emission shift from green (~529 nm) to red (~590 nm). Consequently, mitochondrial depolarization is indicated by a decrease in the red/green fluorescence intensity ratio. JC-1 fluorescence levels were analyzed by flow cytometry to quantitate MMP levels. Additionally, mtDNA was extracted by commercial kit (Biotake, Beijing, China) according to the manufacturer's instructions. Mitochondrial DNA (mtDNA) copy number was detected by qRT-PCR and calculated through delta-delta Ct method; 18srRNA was used as the internal control.

### 2.7. Electron Microscopy

Fresh kidney cortex tissues or mPTCs were fixed in 1.25% glutaraldehyde/0.1 M phosphate buffer and postfixed in 1% OsO_4_/0.1 M phosphate buffer. Ultrathin sections (60 nm) were cut on a microtome, placed on copper grids, stained with uranyl acetate and lead citrate, and examined under an electron microscope (JEOL JEM-1010, Tokyo, Japan).

### 2.8. Masson Trichrome Staining

Harvested kidney tissues from mice were fixed with 4% paraformaldehyde, embedded in paraffin, and sectioned transversely. After dewaxing and gradient ethanol hydration, kidney sections (3 *μ*m) were stained with Weigert's hematoxylin solution for 10 min, rinsed in distilled water for 5 min, stained with Masson ponceau acid fuchsin solution for 10 min, rinsed in distilled water for 2 min, placed in 1% phosphomolybdic acid solution for 5 min, and immersed in aniline blue solution for 5 min. Collagenous matrix was stained blue by Masson staining. The renal pathological changes were observed under microscope (400x magnification).

### 2.9. Analysis of Urine Albumin

Urine samples were centrifuged for 5 min at 12,000 g. Urinary concentrations of albumin were determined using enzyme-linked immunosorbent assay (EIA) kits from Exocell (Philadelphia, PA) according to the manufacturer's instructions.

### 2.10. Statistical Analysis

All data were expressed as the mean ± SD and were analyzed by ANOVA followed by Bonferroni's comparison test. *p* < 0.05 was considered significant.

## 3. Results

### 3.1. TGF-*β*1 Treatment Remarkably Altered Cellular Phenotype in mPTCs

**Figure 1:**
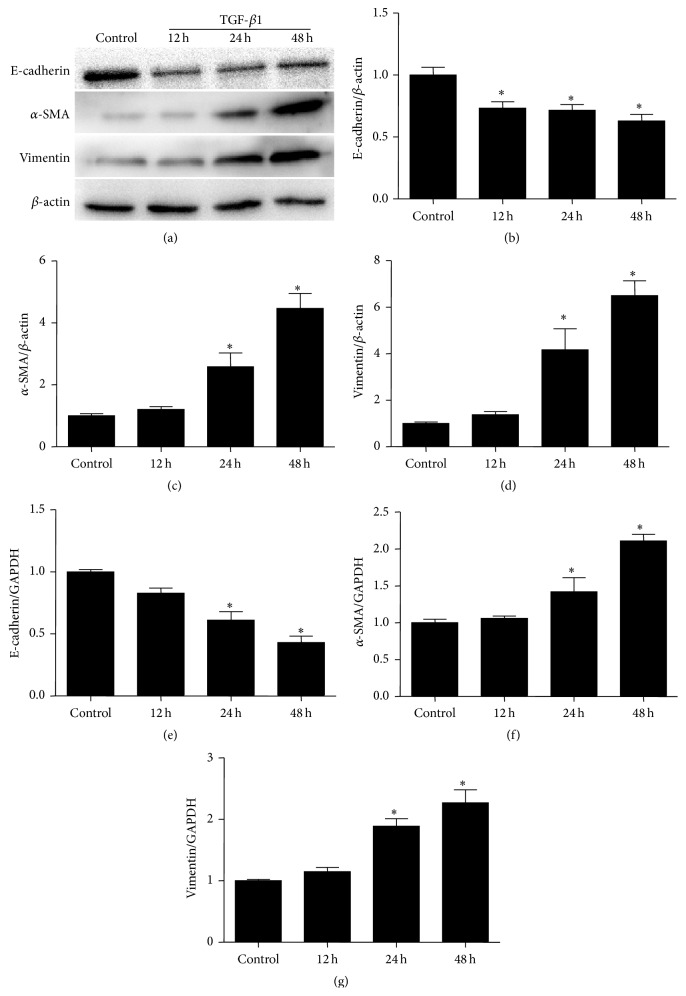
TGF-*β*1 altered cellular phenotype in mPTCs. (a) Western blots of E-cadherin, *α*-SMA, and vimentin. (b–d) Densitometric analysis of Western blots. (e–g) qRT-PCR analysis of E-cadherin (e), *α*-SMA (f), and vimentin (g). All values are means ± SD (*n* = 5 in each group). ^*∗*^ *p* < 0.05 versus control group.

To confirm TGF-*β*1 effect on inducing cellular phenotypic alteration in mPTCs, we treated mPTCs with TGF-*β*1 for 12 h, 24 h, and 48 h and observed the regulation of E-cadherin, vimentin, and *α*-SMA. As shown by Figures 1(a)–1(g), TGF-*β*1 markedly decreased E-cadherin but significantly enhanced vimentin and *α*-SMA at both mRNA and protein levels, indicating a significant alteration of cellular phenotype.

### 3.2. TGF-*β*1 Induced Mitochondrial Dysfunction in mPTCs

**Figure 2:**
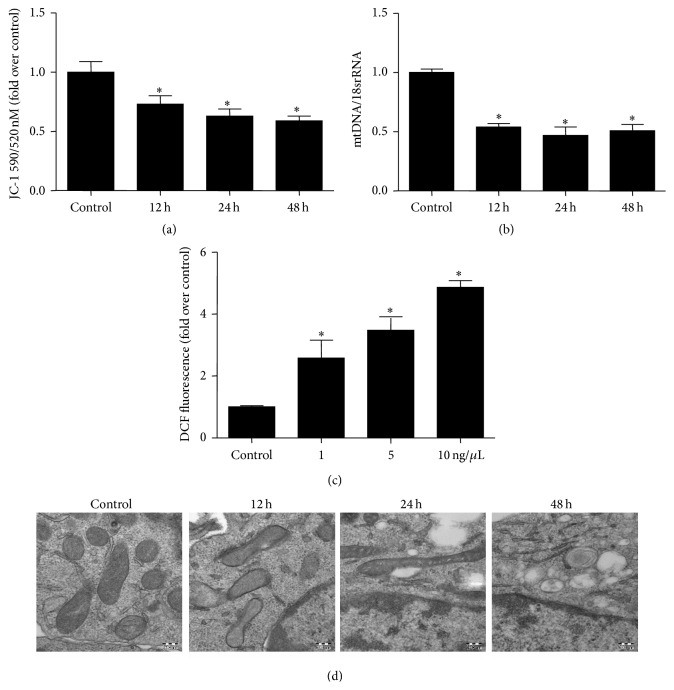
TGF-*β*1 induced mitochondrial dysfunction in mPTCs. (a) Analysis of mitochondrial membrane potential by JC-1 fluorescence dye in mPTCs treated with TGF-*β*1. (b) qRT-PCR analysis of mtDNA copy number in mPTCs treated with TGF-*β*1. (c) Analysis of ROS by DCF fluorescence in mPTCs treated with TGF-*β*1. (d) Electron microscopy analysis of mitochondrial morphology. All values are means ± SD (*n* = 5 in each group). ^*∗*^ *p* < 0.05 versus control group.

To examine the role of TGF-*β*1 in modulating mitochondrial function, mPTCs were treated with TGF-*β*1 and the mitochondrial function was evaluated by measuring MMP, mtDNA copy number, and superoxide production; as shown in Figure 2(a), the fluorescence intensity of JC-1 (5,5′,6,6′-tetrachloro-1), an indicator of MMP, was time-dependently reduced following TGF-*β*1 treatment, indicating a decreased MMP. Meantime, the copy number of mtDNA was also reduced in a time-dependent manner (Figure 2(b)). In contrast, ROS production was elevated as determined by 2′,7′-dichlorofluorescein (DCF) fluorescence (Figure 2(c)). Moreover, TGF-*β*1-treated cells displayed mitochondrial vacuolization and decreased mitochondrial number in mPTCs in a time-dependent manner (Figure 2(d)). These data demonstrated that mitochondrial abnormality occurs in line with cellular phenotypic alteration in response to TGF-*β*1 challenge.

### 3.3. MnTBAP Treatment Prevented Cellular Phenotypic Alteration and Mitochondrial Dysfunction in mPTCs following TGF-*β*1 Challenge

**Figure 3:**
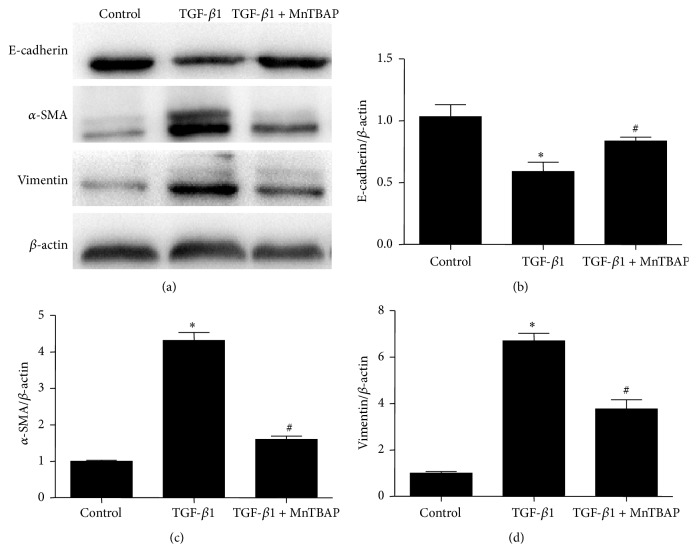
Effects of MnTBAP treatment on protein expressions of E-cadherin, *α*-SMA, and vimentin in mPTCs following TGF-*β*1 treatment. (a) Western blots of E-cadherin, *α*-SMA, and vimentin. (b–d) Densitometric analysis of Western blots. All values are means ± SD (*n* = 5 in each group). _ _^*∗*^ *p* < 0.05 versus control group. _ _^#^ *p* < 0.05 versus TGF-*β*1 group.

**Figure 4:**
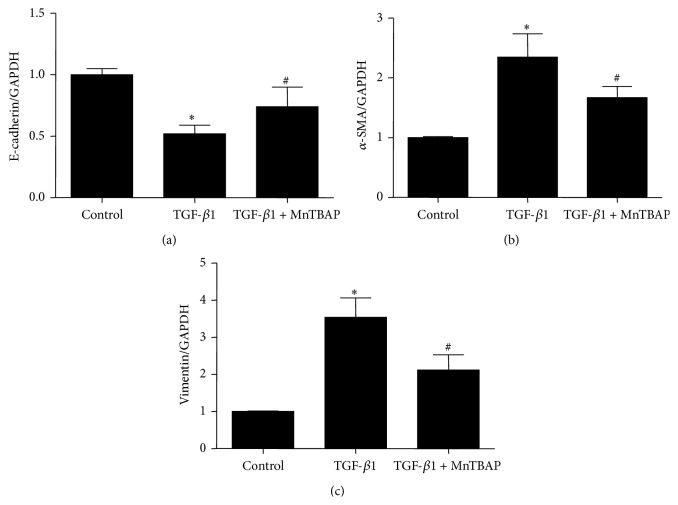
Effects of MnTBAP treatment on mRNA expressions of E-cadherin, *α*-SMA, and vimentin in mPTCs following TGF-*β*1 treatment. (a–c) qRT-PCR analysis of E-cadherin (a), *α*-SMA (b), and vimentin (c). All values are means ± SD (*n* = 5 in each group). _ _^*∗*^ *p* < 0.05 versus control group. _ _^#^ *p* < 0.05 versus TGF-*β*1 group.

**Figure 5:**
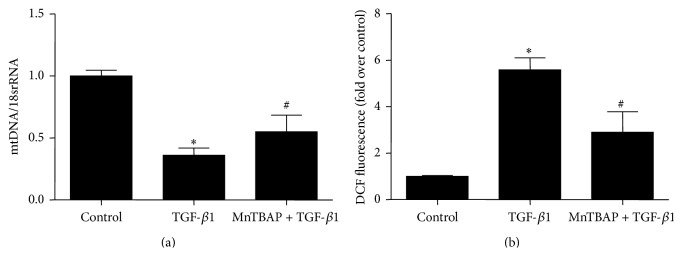
MnTBAP attenuated mitochondrial abnormality induced by TGF-*β*1 in mPTCs. (a) qRT-PCR analysis of mtDNA copy number in mPTCs. (b) Flow cytometry analysis of ROS production by DCF fluorescence in mPTCs. All values are means ± SD (*n* = 5 in each group). _ _^*∗*^ *p* < 0.05 versus control group. _ _^#^ *p* < 0.05 versus TGF-*β*1 group.

To explore whether MnTBAP treatment plays a role in antagonizing fibrotic response, mPTCs were pretreated with MnTBAP followed by TGF-*β*1 administration. As shown in Figures 3(a)–3(d), MnTBAP partially but significantly restored E-cadherin reduction and suppressed the induction of *α*-SMA and vimentin as determined by Western blotting. By qRT-PCR, we observed a similar effect of MnTBAP on regulating these indices at mRNA levels (Figures 4(a)–4(c)). Next, we evaluated mitochondria function and found that MnTBAP pretreatment led to a higher mtDNA copy number and lower ROS production in mPTCs treated with TGF-*β*1 (Figures 5(a) and 5(b)). These data suggested that MnTBAP had a substantial role in preventing TGF-*β*1-induced fibrogenesis possibly via inhibiting mitochondrial oxidative stress.

### 3.4. Kidney Mass Reduction by 5/6 Nx in Mice Caused Tubulointerstitial Fibrosis and Mitochondrial Dysfunction

**Figure 6:**
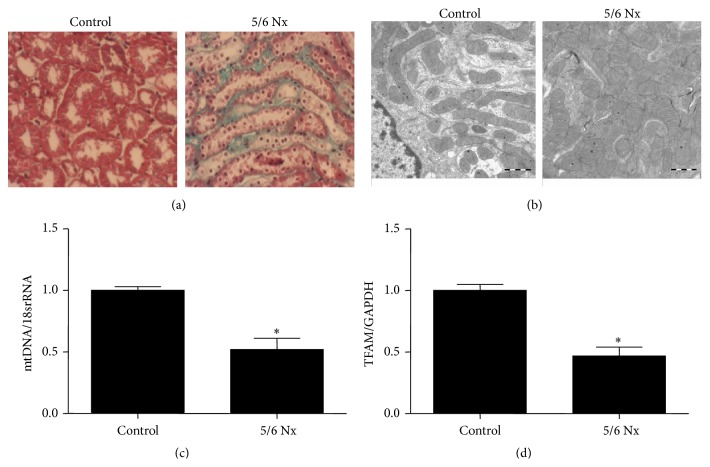
Kidney mass reduction by 5/6 Nx induced renal fibrosis and mitochondrial abnormality. (a) Masson staining. (b) Electron microscopy analysis of mitochondrial morphology in tubular cells. (c) qRT-PCR analysis of mtDNA copy number and TFAM (d) in remnant kidneys. All values are means ± SD (*n* = 5 in each group). _ _^*∗*^ *p* < 0.05 versus control group.

Following 5/6 Nx for 12 weeks, remnant kidney developed significant and extensive tubulointerstitial fibrosis as determined by Masson staining (Figure 6(a)). Meanwhile, mitochondria in tubular cells from fibrotic regions displayed vacuolization and decreased distribution (Figure 6(b)). By qRT-PCR, we detected striking reduction of mtDNA copy number and TFAM mRNA expression in remnant kidneys (Figures 6(c)-6(d)). These data suggested that mitochondrial abnormality occurred in line with renal fibrosis in 5/6 Nx model.

### 3.5. MnTBAP Therapy Attenuated Renal Fibrosis in 5/6 Nx Model

**Figure 7:**
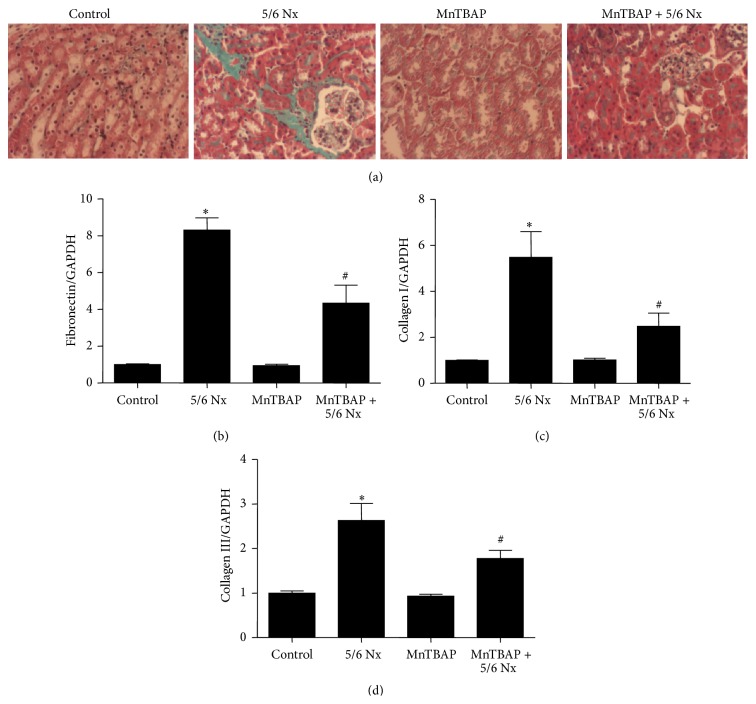
MnTBAP therapy attenuated renal fibrosis in 5/6 Nx mice. (a) Masson staining. (b–d) qRT-PCR analysis of fibronectin (b), collagen I (c), and collagen III (d) in 5/6 Nx mice. All values are means ± SD (*n* = 5 in each group). _ _^*∗*^ *p* < 0.05 versus control group. _ _^#^ *p* < 0.05 versus 5/6 Nx group.

**Figure 8:**
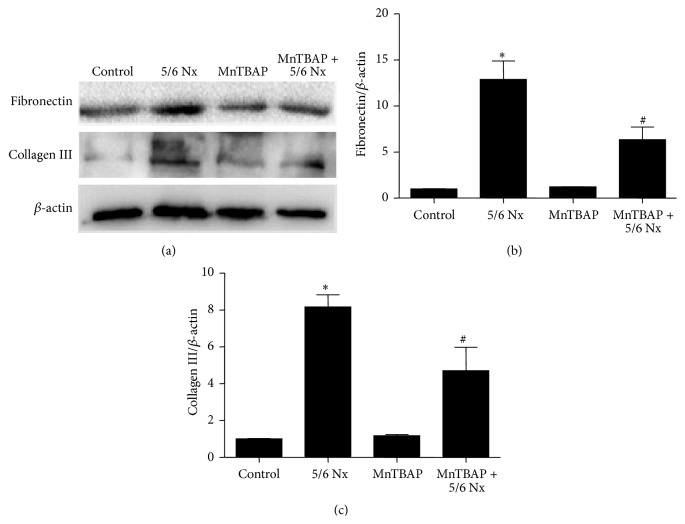
MnTBAP therapy reduced protein expressions of fibronectin and collagen III in remnant kidneys. (a) Western blots of fibronectin and collagen III. (b) Densitometric analysis of Western blots. All values are means ± SD (*n* = 5 in each group). _ _^*∗*^ *p* < 0.05 versus control group. _ _^#^ *p* < 0.05 versus 5/6 Nx group.

To investigate the effect of MnTBAP therapy on renal fibrosis in CKD, the mice were given MnTBAP treatment following 5/6 Nx surgery. By Masson staining, the fibrotic area with blue color in MnTBAP-treated 5/6 Nx mice was reduced as compared to 5/6 Nx controls (Figure 7(a)). By qRT-PCT (Figures 7(b)-7(c)) and Western blotting (Figures 8(a)–8(c)), we further confirmed that MnTBAP significantly ameliorated the deposition of extracellular matrix components including fibronectin, collagen I, and collagen III.

### 3.6. MnTBAP Therapy Ameliorated Proteinuria in 5/6 Nx Model

**Figure 9:**
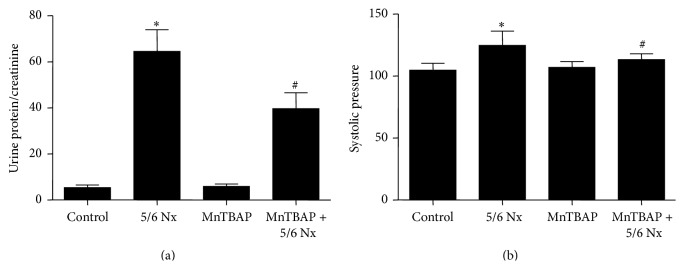
Effects of MnTBAP treatment on proteinuria and blood pressure in 5/6 Nx mice. (a) Urinary albumin excretion. (b) Systolic blood pressure. All values are means ± SD (*n* = 5 in each group). _ _^*∗*^ *p* < 0.05 versus control group. _ _^#^ *p* < 0.05 versus 5/6 Nx group.

Finally, we detected the output of urinary albumin and systolic blood pressure and found that MnTBAP partially improved the severity of proteinuria without affecting CKD-related high blood pressure in 5/6 Nx mice (Figures 9(a)-9(b)).

## 4. Discussion

CRF is characterized by the destruction of a large number of nephrons, as well as the development of renal tubulointerstitial fibrosis. Reduction of renal mass by 5/6 Nx can effectively decrease the number of nephrons, leading to the elevated filtration and drainage in remnant nephrons and impaired endocrine capability. The remaining nephrons in 1/6 kidney are unable to maintain a homeostasis of internal environment, resulting in CRF at last [15]. By now, 5/6 Nx serves as a classic model with the best relevance to human CRF and was widely used to investigate the mechanisms and therapies of CKD and CRF. In this model, renal tubulointerstitial fibrosis is along with the progression of kidney damage and is thought to be a causative factor in the pathological process.

In vitro, TGF-*β*1, a known profibrotic cytokine, induced fibrotic phenotype in mouse renal tubular cells, which was remarkably blunted by MnTBAP, highly suggesting a potential of MnTBAP on antagonizing fibrogenesis. In parallel with the alteration of cellular phenotype, mitochondrial damage also occurred in the tubular cells as evidenced by the altered mitochondrial morphology, reduced MMP and mitochondrial DNA copy number, and increased ROS production. However, following MnTBAP administration, such abnormalities in mitochondria were significantly attenuated. In consideration of the pathogenic role of mitochondrial dysfunction in kidney diseases [16–18], it is reasonable to conclude that the effect of MnTBAP therapy on inhibiting fibrotic response in cells may result from the protection of mitochondrial function to some extent.

To evaluate the in vivo role of MnTBAP in antagonizing renal fibrosis, long term administration of MnTBAP was applied to the mice with 5/6 Nx. Consistent with in vitro results, 5/6 Nx caused remarkable mitochondrial abnormality in tubular cells in line with obvious tubulointerstitial fibrosis. However, 5/6 Nx mice with MnTBAP treatment exhibited significant improvement of renal fibrosis as evidenced by the reduced deposition of extracellular matrix (fibronectin, collagen I, and collagen III). These data provided in vivo evidence showing that MnTBAP could protect against fibrogenesis in renal disease. Targeting mitochondrial oxidative stress might be an effective strategy in retarding the renal fibrosis and disease progression in CKDs.

Besides the antifibrotic action of MnTBAP observed in remnant kidneys, we also found that the severity of proteinuria was significantly ameliorated by MnTBAP treatment. Considering the known role of oxidative stress in kidney diseases [19, 20], we could speculate that the inhibition of mitochondrial oxidative stress and improvement of mitochondrial function could protect podocytes and glomeruli, leading to the attenuation of proteinuria. Moreover, our previous study demonstrated an antihypertensive role of MnTBAP in DOCA-salt hypertension [21], while, in the present study, CKD-related high blood pressure was not affected by MnTBAP treatment possibly due to the distinct pathogenic mechanism of hypertension between models.

In summary, the findings from in vivo and in vitro studies demonstrated an important role of MnTBAP in opposing fibrogenesis in CKDs possibly through attenuating mitochondrial oxidative stress and mitochondrial dysfunction. Thus targeting mitochondrial oxidative stress could serve as a promising strategy in retarding renal fibrosis and disease progression.
